# Tacrolimus versus hydrocortisone in management of atopic dermatitis in children, a randomized controlled double‐blind study: New insights on TARC, CTACK, TSLP, and E‐selectin

**DOI:** 10.1002/iid3.70028

**Published:** 2024-11-26

**Authors:** Ammena Y. Binsaleh, Fedaa A. Kotkata, Mostafa M. Bahaa, Amir O. Hamouda, Mohamad A. El‐Gammal, Aya Ibrahim Elberri, Hend Mostafa Selim, Marwa Ahmed El‐samongy, Manal A. Hamouda, Fatma A. Mokhtar, Sherif Ashraf Fahmy, Thanaa A. Elmasri, Eman I. Elberri

**Affiliations:** ^1^ Department of Pharmacy Practice, College of Pharmacy Princess Nourah bint Abdulrahman University Riyadh Saudi Arabia; ^2^ Department of Clinical Pharmacy, Faculty of Pharmacy Tanta University Tanta Al‐Gharbia Egypt; ^3^ Pharmacy Practice Department, Faculty of Pharmacy Horus University New Damietta Egypt; ^4^ Pharmacology and Biochemistry Department, Faculty of Pharmacy Horus University New Damietta Egypt; ^5^ Department of Zoology, Genetic Engineering and Molecular Biology Division, Faculty of Science Menoufia University Shebin El‐Kom Menoufia Egypt; ^6^ Biochemistry Department, Faculty of Pharmacy Tanta University Tanta Al‐Gharbia Egypt; ^7^ Dermatology and Venerology Department, Faculty of Medicine Tanta University Tanta Al‐Gharbia Egypt; ^8^ Department of Clinical Pharmacy, Faculty of Pharmacy Menoufia University Shebin El‐Kom Menofia Egypt; ^9^ Department of pharmacognosy Faculty of pharmacy, El Saleheya El Gadida University El Saleheya El Gadida Sharkia Egypt; ^10^ Fujairah Research Centre Sakamkam Fujairah UAE; ^11^ Department of Chemistry, School of Life and Medical Sciences University of Hertfordshire Hosted by Global Academic Foundation New Capital Cairo Egypt; ^12^ Pharmacology and Toxicology Department, Faculty of Pharmacy Tanta University Tanta Al‐Gharbia Egypt

**Keywords:** atopic dermatitis, CTACK, E‐selectin, hydrocortisone, tacrolimus, TRAC

## Abstract

**Introduction:**

Atopic dermatitis (AD) is a type of chronic inflammatory disorder that affects all age groups including children. AD is characterized by elevated inflammatory marker levels.

**Aim:**

To assess the safety and effectiveness of topical tacrolimus ointment versus topical hydrocortisone cream in the treatment of pediatric AD by comparing the two treatments' ability to reduce serum cytokines.

**Patients and Methods:**

One hundred AD patients who fulfilled the eligibility criteria completed this clinical study. Two groups of 50 AD patients each were selected from Tanta University's Dermatology Department., Group 1 (the hydrocortisone group) was administered topical hydrocortisone cream for a duration of 4 months. For 4 months, Group 2 was administered tacrolimus topically. Serum levels of thymus and activation regulated chemokine (TARC), cutaneous T cell attractant chemokine (CTAC), interleukin‐10 (IL‐10), interleukin‐6 (IL‐6), E selectin (E‐selectin), and thymic stromal lymphopoietin (TSLP) were measured during an evaluation of the patients by a dermatologist at the beginning and 4 months after the treatment had been started. Children's Dermatology Life Quality Index was used to assess quality of life in these patients.

**Results:**

With the exception of E‐selectin, IL‐6, and IL‐10 (*p* > .05), the tacrolimus group had a significant reduction in TARC, CTACK, TSLP (*p* < .05) when compared to its baseline and when compared to the hydrocortisone group. Both groups showed a significant improvement in quality of life but no significant changes between groups were observed.

**Conclusion:**

In children with AD, tacrolimus reduces inflammatory biomarkers better than hydrocortisone.

## 1 INTRODUCTION

About 15−20% of children worldwide suffer from atopic dermatitis (AD), a chronic inflammatory skin disease manifested by erythema, itch, and eczematous lesions.^1^ Due to its relapsing‐remitting pattern and the possibility of long‐term consequences such skin barrier malfunction and decreased quality of life, managing pediatric AD is extremely difficult.^2^ A family history of other atopic diseases, such as asthma or allergic rhinitis, is typically linked to this kind of dermatitis.^3^

The age of the patient influences how AD presents clinically. An erythematous, papular skin rash that may initially show up on the cheeks and chin is typically the first sign of the condition in infants. When a child scratches, their skin is rough, dry, flaky, cracked, and sometimes bleeds. Adults seem to have more diffuse lesions with erythema.^4^ This condition is divided into two phases: an acute phase, characterized by red, scaly areas on the skin, and a chronic phase, characterized by thicker skin.^5^

The complex and multifaceted pathogenesis of AD includes IgE‐mediated hypersensitivity, alterations in cell‐mediated immune reactions, barrier dysfunction, and environmental factors.^6^ Filaggrin mutations have been associated with severe AD, possibly as a result of increased trans‐epidermal water loss, dehydration, and changes in pH. Additional genetic alterations have also been discovered that could affect the activity of the skin's barrier and cause AD manifestation.^6^

The imbalance of Th2 to Th1 cytokines observed in AD can lead to both IgE‐mediated hypersensitivity and alterations in cell‐mediated immune responses, and these factors appear to be involved in the course of the disease.^7^ In addition, one needs to consider how the environment plays a part in the progression of AD and the effects of harsh detergents, preservatives, airborne formaldehyde, and scents.^8^ The pH of the skin may be adversely altered by the use of harsh alkaline detergents in skin care products, that lead to changes in enzyme production and inflammation.^9^ Both the innate and adaptive immune mechanisms may get activated in response to environmental contaminants.^9^

There is a notable infiltration of CD4‐activated T lymphocytes in the dermis of AD acute lesions.^10^ It is commonly known that the chemokine–chemokine receptor network plays a fundamental role in the accumulation of certain leucocytes that migrate from circulation to inflamed tissue.^11^ Thymus, activation‐regulated chemokine (TARC), and cutaneous T cell‐attracting chemokine (CTACK) are examples of β‐chemokines that are crucial for the skin‐specific recruitment of T cells.^12^

TARC (CCL17) binds to CCR4, a chemokine receptor that is likely necessary for Th2 recruiting and homing in the target organs.^13^ It has been proposed that TARC is released by several cell types, including activated T cells, keratinocytes, endothelial cells, and dendritic cells.^14^ CTACK is produced by keratinocytes. TARC and CTACK may function in a different manner. TARC mainly contributes to the early stage of T cell recruitment by promoting integrin‐dependent adhesion and T cell transendothelial migration, while CTACK is crucial for T cell migration into the skin's top layers.^15^ According to a number of recent studies, TARC and CTACK may be helpful indicators of the severity of the disease in adult AD patients.^16^, ^17^

Topical corticosteroids (TCS), which reduce inflammation through many mechanisms, are the cornerstone of AD treatment, against which other treatments are measured.^18^ Despite TCS's remarkable efficacy, they may also cause striae, acne, rosacea, purpura, contact dermatitis, telangiectasias, and acne locally. Systemic absorption can decrease the hypothalamic‐pituitary‐adrenal axis as well as infections, hyperglycemia, cataracts, glaucoma, and developmental delays in children.^19^ It is essential to investigate alternative therapy options because continued use increases the likelihood of these undesirable effects occurring.

Two topical calcineurin inhibitors (TCI), tacrolimus and pimecrolimus, suppress the immune system and act as immunomodulators to prevent acute flares and lessen the degree of severity of chronic flares.^20^ They prevent T cells from producing inflammatory mediators including tumor necrosis factor alpha (TNF‐α) and calcineurin, which stop T cells from proliferating. TCI is thought to be a workable alternative to TCS since it is more effective and has fewer negative effects.^21^

The purpose of this study was to compare tacrolimus and hydrocortisone's ability to lower the inflammatory markers (TARC, CTACK, TSLP, and E‐selectin) that are often high in AD patients that has not been previously compared in investigations.

## 2 PATIENTS AND METHODS

The research was conducted at Tanta University's Department of Dermatology, Faculty of Medicine, from November 2022 to August 2023. One hundred participants who met the inclusion criteria from the Outpatient Clinic participated in this study. Under approval code 35928/10/2022, the study was granted approval by the Institutional Review Board of Tanta University Faculty of Medicine. The technique and design of the study adhered to the Helsinki Declaration and its 1964 revisions. The possibility of leaving the trial at any moment was disclosed to the patients. Patients or their legal representatives have given written informed consent, indicating that they understand the trial's objectives and risks.

### 2.1 Inclusion criteria

Male or female patients diagnosed between the ages of 5 and 16 using the Hanifin and Rajka criteria^22^ were included. All grades of AD except for very sever ones. Furthermore, the ability and willingness to follow all research guidelines, attend all planned sessions, and complete the study satisfactorily.

### 2.2 Exclusion criteria

Excluded from the study were those receiving biological therapy for inflammatory bowel disease or immune suppressing medications, nonsteroidal anti‐inflammatory drug users, and patients on inhaled or systemic steroids. Additionally, patients with AD receiving systemic therapy for the last 4 weeks, as well as those taking any medications that would alter the biomarkers being analyzed in serum, were excluded from the study.

### 2.3 Study design

Based on serum inflammatory mediators, this study assessed the safety and effectiveness of tacrolimus with hydrocortisone in the treatment of children AD. It was a double‐blinded, randomized, prospective clinical study. Informed consent was obtained from the parents of the children.

In 2022, this clinical trial was registered at ClinicalTrials.gov under the number NCT05607901.

**Figure 1:**
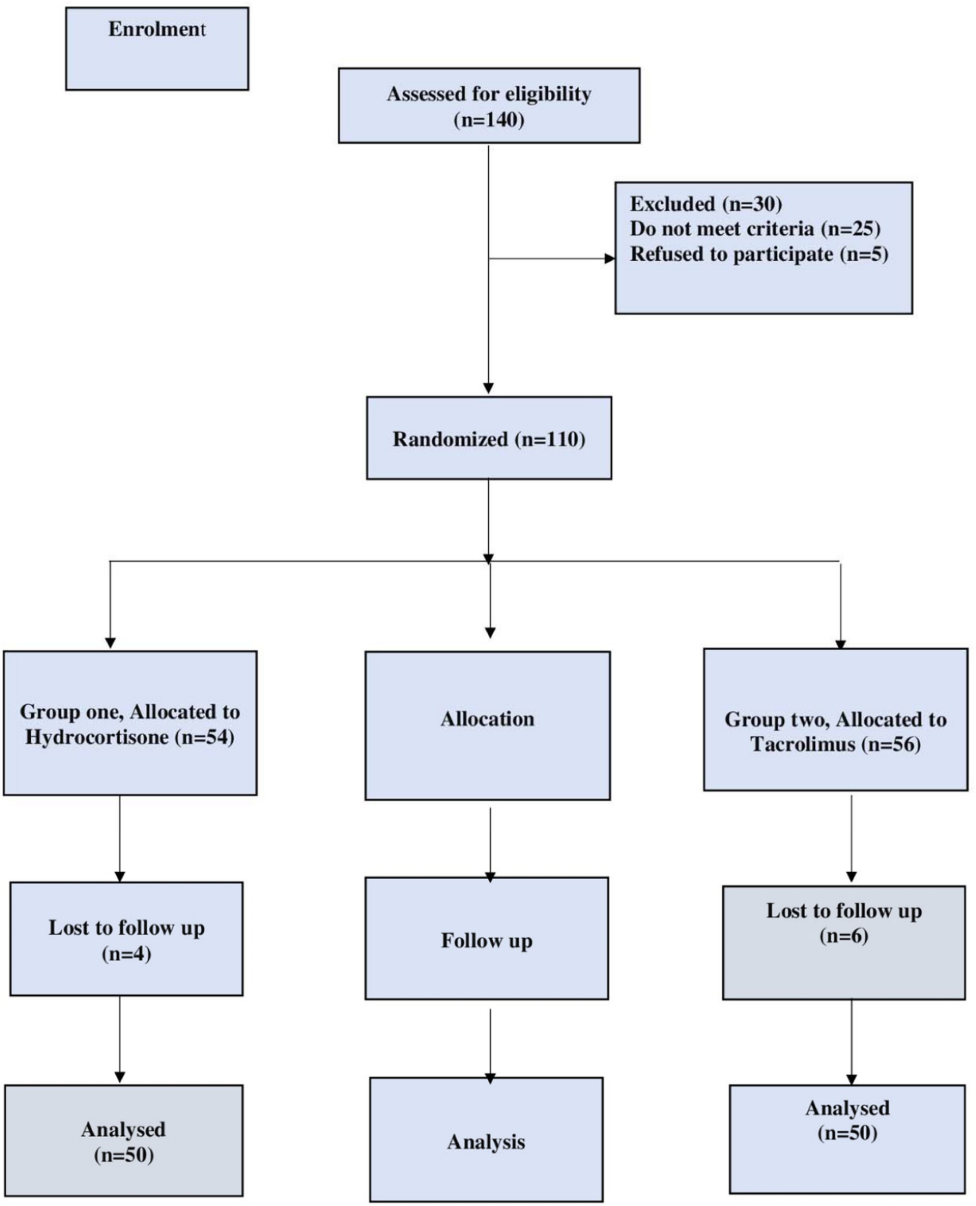
CONSORT diagram showing the flow of the patients during the study.

As per the CONSORT flow diagram presented in Figure 1, the subjects have been divided into two groups at random (*n* = 50). Based on earlier research, the recommended dosage for tacrolimus and hydrocortisone cream.^23^ For the randomization, random permuted blocks were chosen at random using a computer random number generator.

Group 1 (Hydrocortisone group): 50 patients will be treated by applying a thin layer of 1% hydrocortisone cream twice a day to the affected areas for a period of 4 months. (Hydrocort^R^, Multi‐Apex).

Group 2 (Tacrolimus group): 50 patients will get treatment consisting of twice daily application of a thin layer of topical tacrolimus ointment containing 0.03% for a duration of 4 months. (Tarolimus^R^, Andalous Pharma).

### 2.4 Biochemical analysis

Serum samples from each patient were analyzed using Enzyme‐Linked Immunosorbent Assay (ELISA) kits, which were purchased from Sunredio, Shanghai. The kits were used to measure serum levels of interleukin‐10 (IL‐10) (Kit Catalogue No: 201‐12‐0103), IL‐6 (Kit Catalogue No: 201‐12‐0091), E selectin (sE‐selectin) (Kit Catalogue No: 201‐12‐0263), thymic stromal lymphopoietin (TSLP) (Kit Catalogue No: 201‐12‐0427), thymus and activation regulated chemokine (TRAC) (Kit Catalogue No: 201‐12‐0164), cutaneous t cell attractant protein (CTACK) (Kit Catalogue No: 201‐12‐0061).

### 2.5 Sample size calculation and blindness

The sample size was calculated using NCSS, LLC's Power Analysis and Sample Size (PASS) Software, 15th edition (2017), Kaysville, Utah, USA. Based on a previous study,^24^ A large effect size (Cohen' *dz* = 0.8) was hypothesized for the biomarkers that will be used before and after treatments.

Using a two‐sided paired *t*‐test, a sample size of 50 data pairs provides more than 80% power to reject the null hypothesis of zero effect size when the population effect size is .80 and the significance level (*α*) is .05.

### 2.6 Clinical assessment

To determine the degree of dermatitis, each patient had a dermatological examination using the modified Eczema Area and degree Index score.^25^ It is a tool used to measure the extent (area) and severity of atopic eczema. Score of 0 represents no eczema or clear skin, 0.1−1.0 represents practically clear skin, 1.1−7 represents mild illness, 7.1−21 represents moderate disease, 21.1−50 represents severe disease, and more than 51 represents very severe disease.

Additionally, Children's Dermatology Life Quality Index was used to assess quality of life in these patients.^26^

### 2.7 Outcomes

Changes in Children's Dermatology Life Quality Index.^26^ Also, comparing the effect of the tacrolimus and hydrocortisone on serum TRACK, CTACK, TSLP, and E‐selectin.

### 2.8 Statistical analysis

Prism version 9 (GraphPadsoftware, Inc.) was used to conduct the statistical analyses. Continuous variable normal distribution was tested using the Shapiro−Wilk test. The Paired Student's *t*‐test was employed to assess if there was a significant difference within group's pre‐ and post‐therapy states. The unpaired Student's *t*‐test was also used to identify significant differences between groups before and following therapy. The Pearson's correlation test was employed to evaluate the correlation between the measured parameters. The data was displayed as a set of numbers, including the standard deviation (SD), mean, median, and interquartile range. The statistical analysis of categorical data was conducted using the Chi‐square test. The significance level was set at (*p* < .05), and all *p* Values were two‐tailed.

## 3 RESULTS

### 3.1 Baseline demographic data

There were no significant variations in demographic data between the studied groups; age (*p* = .864), sex (*p* = .688), weight (*p* = .081), and height (*p* = .065). Table 1 displayed their baseline statistics.

**Table 1:** Clinical and demographic data in the two study groups.

Parameter	Hydrocortisone group	Tacrolimus group	*p* Value
Age (year)	11.75 (8−13.63)	12. (8−14)	.864*
Sex (M/F)	23/27	25/25	.688**
Height (m^2^)	1.425 (0.88−1.62)	1.6 (0.92−1.68)	.065*
Weight (kg)	47.50 (17.75−60.25)	56.50 (20.75−64.25)	.081*

*Note*: Data are expressed as median, numbers, and interquartile range, Significance at (*p* < .05). Hydrocortisone group: 50 patients received hydrocortisone cream for four months, Tacrolimus group: 50 patients received tacrolimus ointment for four months. *Statistical analysis using Man−Whitney test, **statistical analysis using Chi square test.

Abbreviations: F, female; M, male.

### 3.2 Effect of tacrolimus and hydrocortisone on serum biomarkers

When comparing the two groups using an unpaired *t*‐test, Table 2 showed that there was no significant difference in the baseline values (*p* > .05).

**Table 2:** Analysis of inflammatory biomarkers in the two study groups.

Character	Hydrocortisone group			Tacrolimus group			^b^ *p* Value
	Before treatment	After treatment	^a^ *p* Value	Before treatment	After treatment	^a^ *p* Value	After treatment
IL‐6 (pg/mL)	191.0 ± 21.06	187.9 ± 20.33	.028	192.6 ± 21.33	182.8 ± 16.72	.002	.180
IL‐10 (pg/mL)	133.1 ± 15.98	127.9 ± 15.33	.003	133.5 ± 16.86	128.4 ± 17.18	.0026	.878
CTACK (pg/mL)	1678 ± 51.81	601.3 ± 48.95	.0001	1669 ± 62.01	362.6 ± 16.38	.0001	.0001
E‐selectin (ng/mL)	64.66 ± 10.38	60.68 ± 5.482	.025	62.88 ± 7.199	58.52 ± 9.251	.0003	.159
TRAC (pg/mL)	2825 ± 93.33	2246 ± 76.27	.0001	2790 ± 105.2	1022 ± 37.29	.0001	.0001
TSLP (pg/mL)	2774 ± 112.8	2211 ± 90.97	.0001	2781 ± 108.6	1158 ± 55.55	.0001	.0001

*Note*: Hydrocortisone group: 50 patients received hydrocortisone cream for four months, Tacrolimus group: 50 patients received tacrolimus ointment for four months. Data are expressed as mean ± SD, ^a^significance within groups by paired *t*‐test. ^b^Significance between groups using unpaired *t*‐test. Significance at (*p* < .05).

Abbreviations: CTACK, cutaneous T cell attractant chemokine; IL‐6, interleukin 6, IL‐10, interleukin 10, TRAC, thymus and activation regulated chemokine, TSLP, thymic stromal lymphopoietin,

Regarding group 1, paired *t*‐test showed that there were significant differences in all measured parameters when compared to baseline as follows: IL‐6 (191.0 ± 21.06 vs. 187.9 ± 20.33, *p* = .028), IL‐10 (133.1 ± 15.98 vs. 127.9 ± 15.33, *p* = .003), TARC (2825 ± 93.33 vs. 2246 ± 76.27, *p* = .000), CTACK (1678 ± 51.81 vs. 601.3 ± 48.95, *p* = .000), E‐selectin (64.66 ± 10.38 vs. 60.68 ± 5.482, *p* = .025), and TSLP (2774 ± 112.8 vs. 2211 ± 90.97, *p* = .000) (Table 2).

Regarding group 2, Table 2 revealed that all measured parameters had significant differences from their baseline values as follows: IL‐6 (192.6 ± 21.33 vs. 182.8 ± 16.72, *p* = .002), IL‐10 (133.5 ± 16.86 vs. 128.4 ± 17.18, *p* = .0026), TARC (2790 ± 105.2 vs. 1022 ± 37.29, *p* = .000), CTACK (1669 ± 62.01 vs. 362.6 ± 16.38, *p* = .000), E‐selectin (62.88 ± 7.199 vs. 58.52 ± 9.251, *p* = .0003), and TSLP (2781 ± 108.6 vs. 1158 ± 55.55, *p* = .000) using paired *t*‐test.

Unpaired *t*‐test showed that there were a statistically significant changes in all studied markers after 4 months of intervention, as follows: TARC (*p* = .000), CTACK (*p* = .000), and TSLP (*p* = .0001) except for IL‐6, IL‐10, and E‐selectin.

### 3.3 Effect of studied drugs on children's dermatology life quality index

Regarding hydrocortisone group, Children's Dermatology Life Quality Index was as follow, (20.22 ± 2.854 vs. 9.000 ± 2.129, *p* = .000) using paired *t*‐test.

Regarding tacrolimus group, Children's Dermatology Life Quality Index was as follow, (19.20 ± 2.483 vs. 9.360 ± 1.793, *p* = .000) using paired *t*‐test.

**Figure 2:**
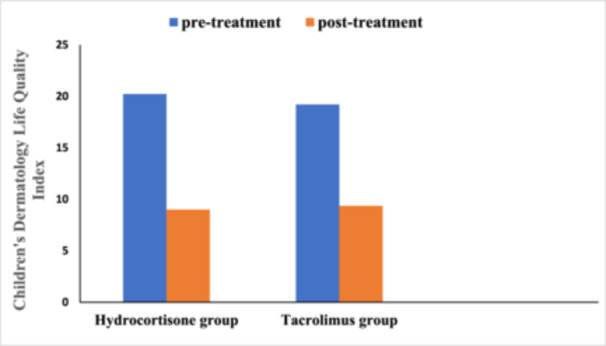
Effect of studied medications on Children's Dermatology Life Quality Index.

Figure 2 showed that both tacrolimus and hydrocortisone group significantly reduced Children's Dermatology Life Quality Index score when compared to their baseline values, but there was nonsignificant difference between the two study groups when compared to after treatment values using unpaired *t*‐test (*p* = .362).

### 3.4 Correlation analysis

There was a significant correlation between Children's Dermatology Life Quality Index and CTACK (*r* = 0.902, *p* = .0001), TARC (*r* = 0.711, *p* = .000) TSLP (*r* = 0.739, *p* = .0001) and a significant correlation between CTACK and TARC (*r* = 0.871, *p* = .0001).

## 4 DISCUSSION

The inflammatory response to AD disease is biphasic, with an initial acute phase dominated by Th2 and Th22 cells and a subsequent chronic phase marked by the simultaneous presence of Th17, Th2 and T helper (Th) cells.^27^ Th2‐derived cytokines and inflammatory mediators secreted by innate immune cells—like mast cells—play a pathogenetic role in the development and exacerbation of skin inflammation in AD lesions. In vitro studies on animal models of AD have shown the innate immune system's involvement in the early stages of the disease, which is probably clinically significant for infants.^28^, ^29^

To our knowledge, this was the first clinical study to compare between tacrolimus and hydrocortisone in AD children regarding serum TARC, CTACK, TSLP, and E‐selectin in children. The current investigation found that the serum TARC level in the tacrolimus group was statistically significantly lower than both its baseline and the hydrocortisone group. Our results are in agreement and in correlation with previous studies.^14^, ^30^, ^31^, ^32^ The hydrocortisone group also showed a significant reduction in serum TARC level when compared to its baseline. These findings are in accordance with previous studies.^14^, ^33^ According to Yasukochi et al., serum TARC levels and topical treatment dosage were strongly correlated. One gram of strong‐rank steroid, or an equivalent dose of steroid/tacrolimus, is required to reduce blood TARC levels by 9.94 pg/mL weekly in people with moderate to severe AD.^30^ Tacrolimus, an anti‐inflammatory medication, may indirectly lower TARC blood levels in AD patients by inhibiting TNF‐α and/or IL‐4‐producing cells from the dermis.^31^ Additionally, corticosteroids reduced lung tissue TARC mRNA expression and TARC protein as well as bronchoalveolar lavage fluid TARC synthesis.^34^ According to a different study, diflorasone diacetate prevented the dinitrochlorobenzene model's induced elevation of TARC.^35^ Since TARC is a particular ligand for CCR4, which causes chemotaxis of CD4 + TH2‐type cells, tacrolimus exerts its suppressive action on TARC indirectly by suppressing Th2 activation.^36^ IFN‐γ and TNF‐α promote the release of TARC, which is produced in keratinocytes.^35^ Thus, tacrolimus decreases TARC levels by downregulation of interferon gamma induced protein (IP‐10) and other cytokines involved in AD.^35^ Tacrolimus prevents TNF‐α from increasing the expression of IP‐10 and monocyte chemoattractant protein‐1 in human colonic myofibroblasts by preventing p38 MAP kinase activity.^37^ These results are supported by the current study and other reports.^37^, ^38^, ^39^

Both tacrolimus group and hydrocortisone group showed a significant decrease in serum CTACK level when compared to their baseline values. Tacrolimus group revealed a significant difference when compared to the hydrocortisone group. These results came in accordance with previous studies that displayed the effect of anti‐inflammatory agent on serum CTACK in AD patients.^40^, ^41^ Keratinocytes produce CTACK, particularly after being stimulated in vitro with TNF‐α and IL‐1β.^42^ When utilizing cortisone and an oral antihistamine, Kakinuma et al. found that patients with AD and psoriasis vulgaris had significantly lower serum CTACK levels.^40^ Tacrolimus exerts its effect on CTACK indirectly by inhibition of TNF‐α, other interleukins, and deactivation of TH2 cells.^43^ In contrast, another study reported that serum CTACK was neither involved nor elevated in a mouse model of dermatitis.^35^

The current study revealed that both tacrolimus and hydrocortisone group showed a significant difference in E‐selectin serum level when compared to baseline value. These results are matched and correlated with several studies that investigated the effect of tacrolimus and cortisone on E‐selectin.^44^, ^45^, ^46^, ^47^, ^48^, ^49^ Dexamethasone showed a suppressive effect on the production of E‐selectin in IL‐1α/TNF‐α‐stimulated endothelial cells after 6 h of stimulation, according to Karim Elias Aziz and Denis Wakefield.^48^ Reduced E‐selectin expression may result in fewer inflammatory cells invading organs harmed by autoimmune disorders.^48^ This dexamethasone's action is mediated through glucocorticoid receptor ligation in humans.^50^ Additionally, cortisone's actions may modify endothelial cells' expression ofcytokines or prostaglandins.^51^ According to Sasakawa et al. and Marcos A et al., tacrolimus prevents peripheral blood mononuclear cells from secreting TNF‐α, therefore decreasing the expression of E‐selectin on vascular endothelial cells.^46^, ^52^

The current study revealed that both tacrolimus and hydrocortisone significantly reduced serum TSLP when compared to their baseline value. Moreover, the tacrolimus group showed a significant decline in TSLP when compared to the hydrocortisone group. These results are matched with several studies.^53^, ^54^, ^55^ TSLP, which is abundantly expressed by keratinocytes in the lesions of AD patients and by bronchial epithelial cells of asthmatic patients, is well established to have a significant role in allergic illnesses.^56^ Dendritic cells that have been stimulated by TSLP release TH2‐recruiting chemokines and cause naive T cells to develop into inflammatory TH2 cells that produce IL‐4, IL‐5, IL‐13, and TNF‐α.^56^ Furthermore, TSLP produce its inflammatory cascade in AD by activating toll‐like receptor pathway.^56^ Antiga et al. reported that topical tacrolimus resulted in a significant reduction in serum toll like receptor in AD.^57^ Others also reported the direct inhibitory effects of tacrolimus on toll like receptors in AD and other diseases.^58^, ^59^, ^60^ Tacrolimus also downregulates TSLP indirectly through the blockade of intracellular calcium and nuclear factor kappa B (NF‑κB) signaling cascade^61^ as TSLP activation is mediated by calcium and NF‑κB pathways.^62^ So, tacrolimus produced its effect on TSLP by indirect mechanisms, such as inhibiting the activity of TH2 cells and toll like receptors. In contrast to our study, several research reported that cortisone were superior to tacrolimus in reducing TSLP.^63^, ^64^ Dexamethasone, but not tacrolimus, inhibits TNF‐induced TSLP production in lesional keratinocytes of an AD model, according to a study by Kazuko Mizuno and his coworkers.^63^ Further studies are required to differentiate these conflicting results.

The current study revealed that both groups significantly reduced serum IL‐6 and IL‐10 in comparison with their baseline values, but there was no statistical significance between the two study groups. These results were matched and correlated with previous studies.^65^, ^66^ In contrast, other studies reported that cortisone was superior to tacrolimus in reducing inflammatory interleukins.^63^, ^67^ Cortisone produces its effect on interleukins and prostaglandins by inhibiting phospholipases and induces biosynthesis of a phospholipase A2 inhibitor which prevents prostaglandin generation.^68^ Tacrolimus reduces interleukins activity by inhibiting TH2 cells as mentioned before.^69^ So, further studies are required to validate these results.

There was a significant correlation between Children's Dermatology Life Quality Index and CTACK, TARC, TSLP, and a significant correlation between CATAC and TARC. These findings are matched and correlated with previous studies.^40^, ^70^, ^71^, ^72^ Certainly, reduction in these inflammatory markers leads to improvement in health‐related quality of life.

Overall, the current study demonstrated that both tacrolimus and hydrocortisone significantly reduced inflammatory markers in children with AD.

## 5 CONCLUSION

This double‐blind, randomized research led us to the conclusion that tacrolimus 0.03% ointment is superior to hydrocortisone cream in terms of reducing inflammatory markers such as TARC, CTACK, TSLP, and E‐selectin in children with AD; nevertheless, there was no difference in terms of the Children's Dermatology Life Quality Index. Generally, tacrolimus is superior to hydrocortisone in the management of AD.

To assess the adverse effect profile, further multicentre, long‐term studies are needed.

## AUTHOR CONTRIBUTIONS

Whether it was in the conceptualization, research design, implementation, data collecting, analysis, and interpretation, or in all of these areas, all authors made a major contribution to the work that was published. They also agreed on the journal to which the article would be submitted, took part in drafting, amending, or critically analyzing the piece, provided final approval for the version that would be published, and agreed to be accountable for all elements of the effort.

## CONFLICT OF INTEREST STATEMENT

The authors declare no conflict of interest.

## ETHICS STATEMENT

The Faculty of Medicine at Tanta University's Institutional Review Board revised the study protocol and gave it their approval for all ethical and scientific considerations. All of the study's participants provided their informed consent.

## Data Availability

Data is provided upon request due to privacy and ethical constraints.
